# Targeting IL-34/MCSF-1R Axis in Colon Cancer

**DOI:** 10.3389/fimmu.2022.917955

**Published:** 2022-06-28

**Authors:** Giovanni Monteleone, Claudia Maresca, Marco Colella, Teresa Pacifico, Daniele Congiu, Edoardo Troncone, Irene Marafini

**Affiliations:** Department of Systems Medicine, University of Rome “TOR VERGATA”, Rome, Italy

**Keywords:** colorectal carcinoma, MCSF-1, tumor-associated macrophages, cancer-associated fibroblasts, tumor microenvironment

## Abstract

Colorectal carcinoma (CRC) is one of the most common neoplasias in the Western world and it is still one of the most deadly cancers worldwide mainly due to the fact that metastatic CRC is not responsive to current pharmacologic treatment. Identification of pathways that sustain CRC cell behaviour could help develop effective therapeutic compounds. A large body of evidence indicates that colon carcinogenesis is a dynamic process in which multiple cell types present in the tumor microenvironment either stimulate or suppress CRC cell growth, survival, and diffusion mainly *via* the production of cytokines. Interleukin-34 (IL-34), a cytokine initially known for its ability to regulate monocyte/macrophage survival and function, is highly produced in human CRC by both cancer cells and non-tumoral cells. IL-34 function is mainly mediated by interaction with the macrophage colony-stimulating factor-1 receptor (MCSF-1R), which is also over-expressed by CRC cells as well as by tumour-associated macrophages (TAMs) and cancer-associated fibroblasts. IL-34-driven MCSF-1R activation triggers several pro-tumoral functions in the colon. In this article, we review the current understanding of the involvement of IL-34 and its receptor in CRC, with particular attention to the available evidence about the IL-34/MCSF-1R axis-mediated regulation of TAMs and the role of IL-34 and MCSF-1R in promoting cancer resistance to chemotherapy and immunotherapy

**Manuscript Contribution to the Field:**

In this review, we highlight the multiple effects of IL-34 and its receptor, macrophage colony-stimulating factor-1 receptor, on the activity of colorectal cancer (CRC) cells and non-tumoral cells, with particular attention to the available data supporting the role of IL-34/MCSF-1R axis in the control of tumor-associated macrophages. The findings summarized in this manuscript could help understand whether targeting IL-34/MCSF-1R can be exploited for therapeutic intervention in CRC.

## Introduction

Colorectal cancer (CRC) is the 3rd most common cancer in the Western world and, despite significant advances in prevention and diagnosis with resection of primary tumor as the first choice, this neoplasia accounts for about 10% of cancer deaths globally (1, 2). This is because in nearly 25% of the cases, diagnosis of CRC is made when cancer has already metastasized and patients with advanced CRC receive little benefit from chemoradiotherapy and immunotherapy (2). About 10% of patients with stage I/II disease and up to 30% of patients with stage III disease develop recurrence after curative resection (3, 4). These later findings support the view that CRC progression is not an autonomous process regarding only cancer cells, but rather a dynamic process incorporating the cross-talk between cancer cells and other immune and non-immune cells present in the tumor microenvironment.

**Figure 1:**
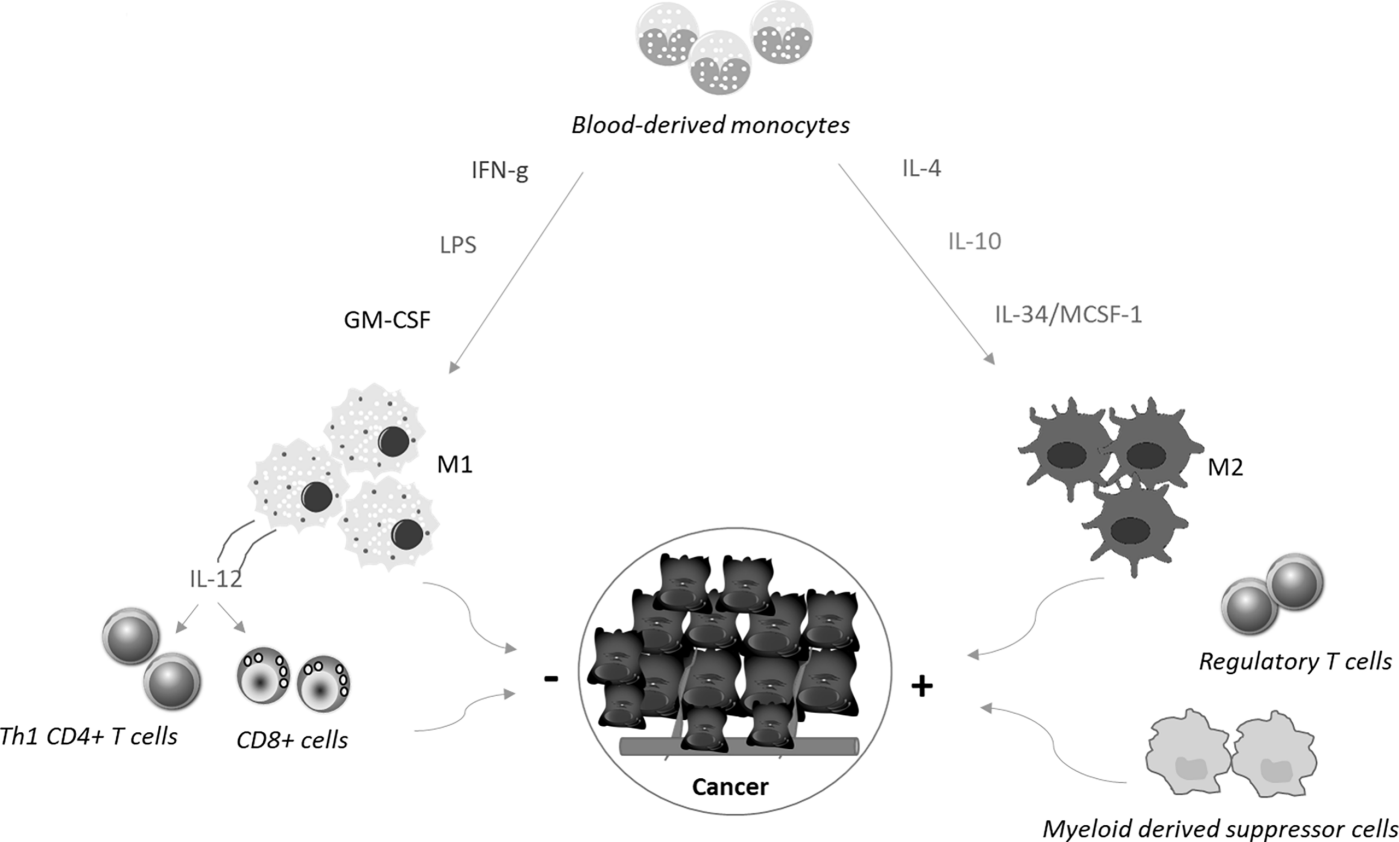
Blood-derived monocytes are recruited to the tumor sites, where cytokines promote their polarization towards M1 or M2 macrophages. M1 macrophages are differentiated in response to the simulation with interferon-γ (IFN−γ), Toll−like receptor ligands, such as lipopolysaccharide (LPS), or granulocyte−macrophage colony−stimulating factor. These cells produce interleukin (IL)-12, which in turn stimulates Th1 cell differentiation and activation of CD8+ T cells. Altogether these cell types limit cancer growth and diffusion. In contrast, together with regulatory T cells and myeloid-derived suppressor cells, M2 macrophages, which are differentiated in response to stimulation with IL−4, IL−10, and IL-34/MCSF-1, contribute to generate a microenvironment that sustains cancer cell behaviour.

Macrophages are the most abundant immune cells in solid tumors, representing up to 50% of all tumor mass (5). They originate mainly from the blood compartment and migrate to the tumor site (monocyte−derived tumor−associated macrophages, TAMs), in response to the action of chemotactic factors produced in the cancer microenvironment (6). Additional macrophage categories present in the tumor microenvironment include tissue−resident macrophages and myeloid-derived suppressor cells (7). Macrophages can change their phenotype and status, and both tumor-killing and tumor-promoting macrophage subpopulations can be present in the tumor mass during the various phases of the neoplastic progression (8, 9). On the basis of the activating stimuli and their prevalent function, macrophages can be categorized into classically activated, pro-inflammatory M1 macrophages and alternatively activated, anti-inflammatory M2 macrophages (10) (**Figure 1**). M1 macrophages exert mainly anti-tumor function, while M2 macrophages contribute to malignancy through production of tumor and angiogenic growth factors, extracellular matrix remodeling, and immunosuppression and high numbers of these cells often correlate with a bad prognosis and therapeutic resistance (11–13). However, recent single cell sequencing approaches of various cancers and the corresponding non-tumoral tissues of cancer patients have shown that macrophage subtypes present in tumors, including CRC, do not exactly match the criteria of dichotomous M1/M2 phenotypes (14–16).

In recent years, studies from several laboratories have shown that interleukin-34 (IL-34), a cytokine initially known to regulate the survival and function of monocytes/macrophages (17), is over-expressed in many cancers, where it regulates several cancer cell functions (18–20). In this article, we review the current understanding of the role of IL-34 and its receptor, macrophage colony-stimulating factor-1 receptor (MCSF-1R, also termed CSF-1R or FMS), in CRC, with particular attention to the available evidence about the IL-34/MCSF-1R axis-mediated regulation of TAMs and the role of IL-34 and MCSF-1R in promoting cancer resistance to chemotherapy and immunotherapy.

## IL-34-Driven MCSF1-R Signalling

IL-34 was discovered by Lin and colleagues, who showed the ability of the cytokine to bind human CD14+ monocytes and increase their viability (17). The authors demonstrated that interaction of purified IL-34 with a monocyte cell line was blocked by co-incubation of IL-34 with the extracellular domain of MCSF-1R, a class III receptor tyrosine kinase, and the IL-34-mediated enhancement of monocyte viability was independent of MCSF-1, another ligand of MCSF-1R (17). IL‐34 and M-CSF‐1 bind to overlapping regions of M-CSF-1R thus stimulating autophosphorylation of the receptor (21), even though the two ligands activate distinct signalling pathways depending on the hydrophobic/hydrophilic interaction of each cytokine with the receptor (22–27). In particular, binding of IL-34 to M-CSF-1-R is mediated by hydrophobic interaction, which promotes a stronger intracellular signalling than that induced by the hydrophilic interaction of MCSF-1 with M-CSF-1R (21). Depending on the cell type analysed, IL-34 and MCSF-1 can activate several signalling pathways [(e.g. p38 mitogen-activated protein kinase (MAPK), c-Jun N-terminal kinase (JNK), extracellular signal-regulated protein kinases 1 and 2 (ERK1/2), nuclear factor kappa-light-chain-enhancer of activated B cells (NF-κB), phosphoinositide 3-kinase (PI3K)/AKT, Janus kinase (JAK), signal transducer and activator of transcription (STAT)3] (21, 28–31), thus contributing to explain the non-redundant and sometimes divergent roles of the two cytokines on some macrophage functions. For example, IL-34-stimulated macrophages produced lesser MCP-1 but more eotaxin-2 and have reduced capacity for bacteria phagocytosis as compared to MCSF-1-stimulated macrophages (21, 32). Although both IL-34 and MCSF-1 have the same ability to induce IL-10 production by macrophages, IL-34-derived macrophages synthesise less IL-12 than MCSF-1-stimulated macrophages (33). However, in response to inflammatory stimuli, such as lipopolysaccharide and interferon (IFN)-*γ*, production of IL-10 and CXCL11 is higher in IL-34-treated macrophages than in M-CSF-1-stimulated macrophages, while in response to regulatory stimuli, such as IL-4, IL-34-treated macrophages produce more CCL17 and CCL22 and less IL-10 than M-CSF-1-differentiated macrophages (34). In human monocytes, IL-34 can activate caspase-3/8 and promote autophagy through an AMP-activated protein kinase-UNC-51-like Kinase 1-dependent mechanism (34). The functional differences between IL-34 and MCSF-1 could be also rely on the fact that, unlike M-CSF-1, which only interacts with MCSF-1R, IL-34 can bind two additional receptors, namely receptor-type protein-tyrosine phosphatase zeta (PTP-ζ) and syndecan-1 (also known as CD138) (21, 34).

## IL-34 Regulates Positively Colorectal Cancer Cell Growth

**Figure 2:**
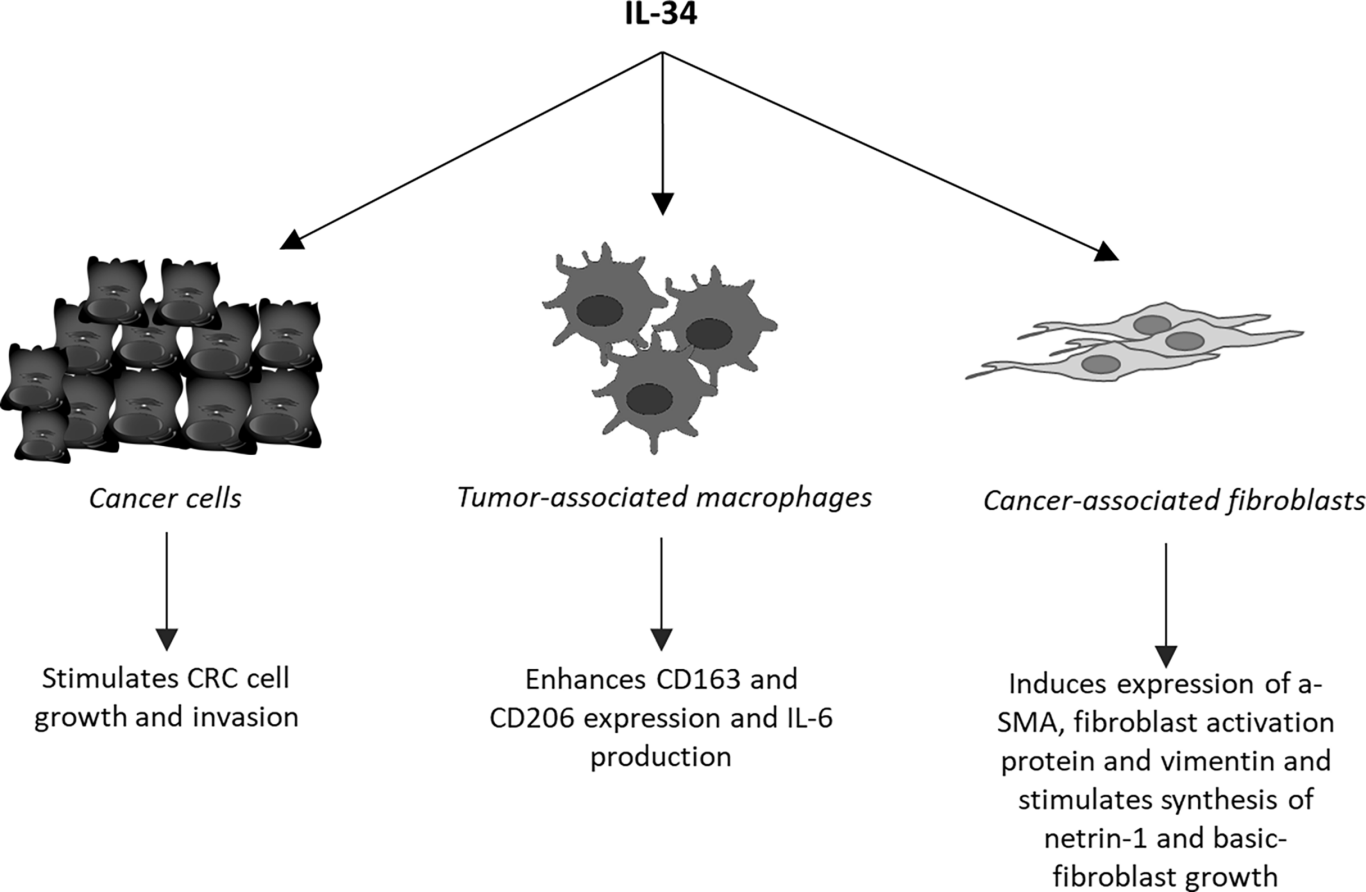
Schematic view of the cell targets and biological effects of interleukin-34 (IL-34) in human colorectal cancer.

Unlike MCSF-1, which is widely expressed in the body under physiological conditions, IL-34 is highly expressed in the brain and skin (35). Nonetheless, IL-34 is produced by many tumor cell types, including CRC cells (18). For instance, Kobayashi and colleagues documented IL-34 expression in various CRC cell lines and CRC tissues from a cohort of Japanese patients and showed that high expression of IL-34 correlated with poor survival of the patients (36). Similar findings were seen in a cohort of CRC patients registered at The Cancer Genome Atlas (37). In contrast, Wang and co-workers showed that reduced expression of IL-34 RNA transcripts associated with poor survival in a cohort of 55 CRC patients (38). By real-time PCR and Western blotting of mucosal samples taken from tumoral and non-tumoral areas of CRC patients and normal controls, we showed that IL-34 was highly expressed in human CRC (39). By immunohistochemistry, we also demonstrated that IL-34 was mostly produced by cancer cells and to lesser extent by tumor-infiltrating mononuclear cells. MCSF-1 is also highly produced by CRC cells and its content correlates with macrophages infiltration (40). Surprisingly however, both the elevated levels of MCSF-1 and macrophage infiltration correlated with the tumor-node-metastasis stage of CRC and associated with improved survival of patients (40). Our immunostaining studies documented over-expression of MCSFR-1 in the tumor areas as compared to the non-tumor areas of CRC patients. Interestingly, both CRC cells and tumor-associated lamina propria mononuclear cells were strongly positive for this receptor, while expression of PTP-ζ was not up-regulated in CRC (39). Treatment of DLD1, a CRC cell line, with exogenous IL-34, but not with MCSF-1, activated ERK1/2 MAP kinase pathway thus resulting in enhanced cell proliferation and migration (**Figure 2**) (39). Consistently, knockdown of IL-34 in DLD-1 with an antisense oligonucleotide (ASO) abrogated ERK1/2 activation thus inhibiting cell growth (39). Overall, these findings indicate that CRC cells produce both IL-34 and MCSF-1 and suggest that these cytokines regulate differently CRC cell growth.

## Role of IL-34/MCSF-1R Axis in the Differentiation and Function of Tumor-Associated Macrophages

As pointed-out above, tumor mass contains a large number of regulatory macrophages that can promote cancer cell growth and diffusion and abrogate the host immune response against cancer cells. It is also known that molecules released from cancer cells following chemotherapy or immunotherapy may enhance cancer cell resistance through modulating the macrophage differentiation and function. One of such molecules could be IL-34. Support to this hypothesis comes from the demonstration that the *in vitro* generated, chemotherapy-resistant human lung cancer cell line, A549, and the corresponding *in vivo* chemo-resistant cancer cells produced IL-34 and induced a monocyte-derived immunosuppressive macrophage population (41). Targeting IL-34 in chemoresistant tumors resulted in a marked inhibition of tumor growth when accompanied with chemotherapy (41). Studies in ovarian cancer have convincingly shown that IL-34 facilitated differentiation of monocytes into IL-10-producing, immunoregulatory macrophages showing similarities to TAMs (33, 42). It has been also demonstrated that IL-34-stimulated macrophages promoted differentiation of CCR4+ CCR6+ CD161+ Th17 cells and IL-34 expression associated with increased infiltration and function of M2 in various cancers (43). In CRC, IL-34 expression correlated with the content of CD163, a marker of TAMs and our data indicated that tumour-infiltrating cells responded to IL-34 by up-regulating not only CD163 but also CD206, another marker of M2, and IL-6, a cytokine that activates proliferative and survival signals in CRC cells (**Figure 2**) (13, 44). These findings well fit with the *in vivo* demonstration that administration of RG7155, a monoclonal antibody that inhibits MCSF-1R activation, to CRC patients led to striking reductions of CSF-1R+CD163+ macrophages in tumor tissues (45). Moreover, Lee K-H and colleagues showed that oral administration of an active and selective MCSF-1R inhibitor to C57BL/6 mice bearing the MC38 murine colon tumor increased the M1/M2 macrophage ratio and the number of cytotoxic CD8+ T-cells within the tumor, with the downstream effect of delaying the tumor growth (46). Altogether these data suggest that the pro-tumorigenic effect of IL-34/MCSF-1R axis in the colon is, at least, in part mediated by induction of immunosuppressive TAMs.

A somehow different scenario emerges from studies assessing the effects of CRC cell-derived MCSF-1 on the properties of tumor-infiltrated macrophages. Wang and colleagues injected recombinant adenovirus vector expressing human MCSF-1 (Ad-hMCSF-1) into the right tumor of nude mice, which were bearing HT-29 CRC xenografts on bilateral legs. The injection of Ad-hMCSF-1 markedly increased the number of tumor-infiltrated macrophages and partially suppressed tumor growth (40). Along the same line is the demonstration that in syngeneic mouse tumor models, treatment of mice with anti-MCSF-1R preferentially depleted macrophages with an inflammatory signature but spared macrophage populations that in mouse and human express pro-angiogenic/tumorigenic genes (15). Thus, it is conceivable that activation of MCSF-1R by either IL-34 or MCSF-1 can differently modulate the differentiation of TAMs thereby resulting in diverging effects on CRC progression.

## IL-34/MCSF-1R Axis and Cancer Immunotherapy

Recent advances in understanding the roles of immune checkpoints in allowing tumors to circumvent the immune system have paved the way for the development of drugs targeting programmed death-1 (PD-1) and/or cytotoxic T-lymphocyte associated protein-4 (CTLA-4), which have fundamentally changed oncology practice (47). Antibody blockade of these immune checkpoints enhances the function of antitumor T cells, at least in part, by relieving inhibition of the T cell costimulatory receptor CD28 (48). Nevertheless, the durable responses induced by PD-1 or PD-L1 blockade alone can be limited in patients with CRC and the poor therapeutic effects have been linked to tumor-intrinsic or -extrinsic mechanisms for escaping immune surveillance (49).

By using molecular markers, CRC can be divided in two main forms, which exhibit different response to immunotherapy. Specifically, high-level microsatellite instability (MSI)/deficient mismatch repair (MMR) is associated with the synthesis of cancer neoantigens by increased tumor mutation burden and increased infiltration of T cells in the tumor microenvironment. Importantly, this form of CRC, which can be seen in nearly 15% of stages I-III CRC and 4% of stage IV CRC, responds well to Pembrolizumab (an anti-PD-1 inhibitor) (50). In contrast, the majority of CRC have microsatellite stable/proficient MMR disease associated with a poor response to immunotherapy. Such poor response can be explained by the immune-exclusive tumor microenvironment, which is associated with up-regulation of WNT signaling and low synthesis of tumor neoantigens (50–52). In this context, the remarkable infiltration of the tumor mass with M2-biased TAMs, myeloid-derived suppressor cells, and regulatory T cells, and production of various metabolic and inflammatory mediators in the tumor microenvironment are supposed to play a role in the resistance to immunotherapy (53–55). This has boosted intensive research aimed at identifying other molecules/pathways that may be targeted alone or together with existing immunotherapies. Among several therapeutic candidates, IL-34 and MCSF-1R have gained attention because these molecules regulate the survival, proliferation, and functions of TAMs with enhanced immunosuppressive activities (20). Shi and colleagues (56) treated mice bearing the CT26 and MC38 colon tumors with a combination of anti-PD-1 antibody, PLX3397 (an oral tyrosine kinase inhibitor of MCF-1R) and oncolytic viruses (OVs), which are known to increase T cell infiltration of tumor due to their advantages to selectively infect and kill tumor cells. The authors showed that the triple treatment reprogrammed the immunosuppressive tumor microenvironment by increasing the number of T cells in the tumor and augmenting anti-tumor CD8+ T cell function. This combination therapy synergistically conferred tumor control and prolonged the survival of mice. As expected, treatment with PLX3397 reduced CD206-expressing TAMs, thus boosting the anti-PD-1 therapy. Indeed, the combination of PLX3397 and anti-PD-1 exhibited more effective tumor control as compared to that seen in mice receiving the single treatment. The therapeutic effects of PLX3397 alone were poor as well as was the monotherapy with anti-PD-1 or OVs. These findings support previous studies showing the limited efficacy of single treatment with MCSF-1R inhibitors in animal models and human cancers (57, 58) as well the therapeutic effects seen when MCSF-1R inhibitors are combined with chemotherapy or immune checkpoint blockade (59, 60).

Recently, Hama et al. inoculated BALB/c mice with tumor CT26 cells either deficient or over-expressing IL-34 and then treated the animals with anti-PD-1 antibody. They showed that IL-34-knockout tumors exhibited a better response when treated with anti-PD-1 antibody whereas the significant effect of PD-1 blockade was abrogated by the existence of IL-34 secreted by tumor cells. Next-generation sequencing analysis and gene ontology analysis indicated that the clusters associated with immune cell response, including “T cell receptor signaling pathway,” “antigen processing and presentation,” and “cytokine-cytokine receptor interaction,” were enriched in the group inoculated with IL-34-deficient CT26 cells and treated with anti-PD-1 antibody. Those mice exhibited also up-regulation of several genes associated with T cell accumulation (i.e. CD3e, CD4, CD8a), inflammation (i.e. TNF, IFN and CXCL19), and M1-macrophages (i.e. CD86, CIITA, NOS2) while M2 macrophage-associated genes (i.e. MRC1, Chi3l3, Arg1) were reduced (61). In further experiments, the authors treated mice bearing tumors generated by IL-34-over-expressing CT26 cells with anti-CTLA-4 antibody and/or anti-PD-1 antibody and/or anti-IL-34 antibody. PD-1 and CTLA-4 combination therapy induced substantial tumor suppression, which was markedly enhanced by anti-IL-34 treatment (61).

Collectively, these findings indicate that IL-34, produced by cancer cells as well as additional cell types present in the tumor mass, interferes with immunotherapy by limiting T cell accumulation and favouring the anti-inflammatory environment.

## IL-34 and Cancer-Associated Fibroblasts

Cancer microenvironment contains also high number of fibroblasts (cancer-associated fibroblasts, CAFs), which facilitate recruitment and dictate the fate of infiltrated monocytes towards a pro-tumorigenic macrophage population (62). In addition, CAFs promote CRC growth and progression, resistance to chemotherapy and relapse of cancer through the synthesis of various molecules targeting the neoplastic cells (63, 64). In human CRC, CAFs express both M-CSFR-1 and PTP-ζ and produce high levels of IL-34 as compared to fibroblasts isolated from the normal, adjacent colonic mucosa of the same CRC patients (65). Stimulation of normal colonic fibroblasts with recombinant IL-34 increases the expression of typical markers of CAFs, such α-SMA, fibroblast activation protein and vimentin, and enhances their proliferation, while knockdown of IL-34 expression in CAFs reduces the expression of these markers and proliferation (**Figure 2**) (65). Moreover, inhibition of IL-34 in CAFs reduces the ability of such cells to promote CRC cell growth and migration, raising the possibility that IL-34-stimulated CAFs synthesize factors regulating CRC cell behaviour. Among these, netrin-1 and basic fibroblast growth factor (b-FGF) are induced by IL-34 (**Figure 2**).

## Conclusions

Accumulating evidence supports the view that IL-34-driven activation of MCSF-1R in different cell types present in the CRC mass triggers signalling pathways that sustain either directly or indirectly CRC cell growth, survival and diffusion as well as resistance to anti-tumor therapeutics. The possibility to use IL-34 or MCSF-1R inhibitors to block the pro-tumorigenic effects of this axis could thus open up a challenging opportunity for a new treatment option in CRC (39, 44, 65). However, pre-clinical work in mouse models of CRC indicates that blockade of IL-34-associated MCSF-1R pathway as monotherapy provides minimal therapeutic benefit despite such a treatment associates with marked reduction of the number of TAMs. This could rely on the fact that additional compensatory mechanisms may limit the anti-tumor activity of IL-34/MCSF-1R blockers, including treatment-induced recruitment of regulatory T cells or myeloid-derived suppressor cells. Indeed, it is known that genetic ablation of MCSF-1 in CRC cells reduces the influx of immunosuppressive MCSF-1R-expressing TAMs within tumors thus resulting in an increase of Foxp3+ regulatory T cells that limit the attack of CD8+ T cells on tumors (66). It has also been reported that MCSF-1R inhibitors alter CAF-derived secretion of CXCL1, thereby attracting pro-tumorigenic myeloid-derived suppressor cells and resulting in poor efficacy. Interestingly, combined inhibition of MCSF1-R and CXCR2 blocks myeloid-derived suppressor cell recruitment and reduces tumor growth, which is further improved by the addition of anti-PD-1 (67). It is also plausible that, in the presence of IL-34/MCSF-1R inhibitors, CRC can activate alternative signals for recruitment, proliferation and/or survival of suppressive macrophages (68, 69).

In contrast to the lack of anti-tumor effect of IL-34 or MCSF-1R inhibitor alone, it seems that each of these compounds may enhance the effect of immunotherapy of chemotherapy (41), thus suggesting a promising opportunity for therapeutic intervention in patients with CRC.

Future work will tell us whether the high expression of IL-34 in CRC tissue is paralleled by high circulating levels of the cytokine and whether IL-34 may serve as a prognostic biomarker in this neoplasia. It remains also to clarify whether the pro-tumorigenic effects of IL-34 here described are in part mediated by other molecules produced by both immune and non-immune cells in response to IL-34 stimulation. For example, in the gut, IL-34 stimulates immune cells to make TNF (23), a cytokine exerting proliferative effects on CRC cells (70). Moreover, IL-34 regulates the activity of regulatory T cells (71) and stimulates macrophages to switch non-Th17 committed memory CD4(+) T cells into Th17 cells (72), with the downstream effect of enhancing CRC cell growth and migration (73).

## Author Contributions

GM searched literature for relevant articles and wrote the article. CM, MC, TP, DC, ET and IM searched literature for relevant articles and revised the manuscript. All authors contributed to the article and approved the submitted version.

## Funding

The study was supported by the Associazione Italiana per la Ricerca sul Cancro (IG2016-19223).

## Conflict of Interest

GM has served as an advisory board member for ABBVIE and First Wave BioPharma. IM has served as a speaker for Janssen.

The remaining authors declare that the research was conducted in the absence of any commercial or financial relationships that could be construed as a potential conflict of interest.

## Publisher’s Note

All claims expressed in this article are solely those of the authors and do not necessarily represent those of their affiliated organizations, or those of the publisher, the editors and the reviewers. Any product that may be evaluated in this article, or claim that may be made by its manufacturer, is not guaranteed or endorsed by the publisher.
